# Parental Sensitivity and Responsiveness as Mediators Between Postpartum Mental Health and Bonding in Mothers and Fathers

**DOI:** 10.3389/fpsyt.2021.723418

**Published:** 2021-09-01

**Authors:** Sandra Nakić Radoš

**Affiliations:** Department of Psychology, Catholic University of Croatia, Zagreb, Croatia

**Keywords:** postnatal depression, anxiety, stress, responsiveness, fathers, mother-infant bonding, maternal sensitivity

## Abstract

**Background:** There is a lack of studies that examine the complex relationship between parental mental health, parental sensitivity and responsiveness, and parent-infant bonding. This study aimed to test whether parental sensitivity and responsiveness were mediators between postpartum mental health (depression, anxiety, and stress) and parent-infant bonding in mothers and fathers.

**Method:** Mothers (*n* = 427) and fathers (*n* = 170) of infants aged up to 1-year-old participated in an online study. The parents completed questionnaires on depression (Edinburgh Postnatal Depression Scale, EPDS), anxiety and stress (Depression, Anxiety, and Stress Scale, DASS-21). Parent-infant bonding was measured by Postpartum Bonding Questionnaire (PBQ) that has three components: Impaired bonding (PBQ1), Anxiety about care and parental distress (PBQ2), and Lack of enjoyment and affection with infant (PB3Q). Parental sensitivity was measured as the number of correct recognitions of infant facial expressions (City Infant Faces Database, CIFD). Responsiveness was measured as a self-report with two subscales of responsiveness and non-responsiveness (Maternal Infant Responsiveness Instrument, MIRI).

**Results:** The path analysis showed that the model had a good fit to the data. Parental sex was a significant moderator, indicating different paths in mothers and fathers. In mothers, responsiveness and non-responsiveness were significant mediators between depression symptoms and three dimensions of bonding. In fathers, only non-responsiveness was a significant mediator between anxiety and PBQ3. Although recognizing infant facial expressions directly affected PBQ3 in mothers (but not in fathers), it was not a significant mediator between mental health and bonding.

**Conclusion:** Higher levels of parental mental health problems (depression and anxiety) were associated with lower levels of parental responsiveness, which is, in turn, related to poor parent-infant bonding. Prevention and intervention programs should be offered for both mothers and fathers, focusing on postpartum mental health promotion and enhancing responsiveness in infant care.

## Introduction

Maternal sensitivity and responsiveness have been identified as crucial for secure infant attachment. Maternal sensitivity seems to be a stable maternal characteristic during infancy (1). It refers to the maternal ability to perceive the infant cues and signals, interpret them correctly, and respond to them timely and adequately (2–4). These iterative processes in mother-infant interactions are essential for infant development as infants learn that their actions affect the environment, especially the secure figure, which gives them a sense of efficacy. Consequently, an infant who feels secure will explore the environment more, which will increase their socio-emotional and cognitive competencies (2).

Shin et al. (5) pointed out that the conception of maternal sensitivity has changed over time. Based on their conceptual analysis, four aspects of maternal sensitivity have been pointed out. These refer to maternal sensitivity as (i) dynamic process, (ii) including reciprocal exchanges between mother and infant, (iii) contingent with infant's previous behavior, and (iv) including appropriate responses based on specific infant cues. Maternal responsiveness is one aspect of sensitivity (5) and refers to maternal prompt and frequent responses to the infant's cues about physical and emotional states (3).

Shin et al. (5) provided a conceptual structure of maternal sensitivity describing its antecedents, affecting factors and consequences. Antecedents are described as maternal identity or identification of self as a mother, and of course, the infant's needs and cues upon which mother will express her sensitivity. One of the consequences of maternal sensitivity is the development of secure mother-infant relationships and higher quality of infant-to-mother attachment. Indeed, there is a bulk of literature showing the association between maternal sensitivity and secure attachment in infancy (1, 6), early childhood (7), and young adulthood (6).

Bonding is sometimes erroneously used as a synonym with attachment (8–10). However, the former can be defined as the maternal feelings and thoughts about the infant (9, 11), while attachment refers to the relationship between the child and the parent and makes the child feel secured (12). Furthermore, the methods of measures differ between the two, with questionnaires to measure bonding (13) and the observational method of Strange Situation Task (14) as a gold standard to measure attachment. As a strong mother-infant relationship, bonding is considered crucial for postpartum development (15). A recent literature review has also shown that maternal sensitivity is sometimes used as a synonym for bonding (16). However, although these are different constructs, there is a lack of studies looking at maternal sensitivity and responsiveness in relation to mother-infant bonding.

As affecting factors on maternal sensitivity, Shin et al. (5) distinguished some positive, such as social support and high self-esteem, and negative factors, such as maternal depression, stress, and anxiety. Maternal mental health in the postpartum period can be seriously challenged, with one in three primiparous women having symptoms of depression, anxiety or stress (17). A recent meta-analysis revealed that around 17% of healthy women report postpartum depression (18) and 8–10% report one or more anxiety disorders (19, 20). Also, comorbidity between postpartum depression and anxiety has been established (21–23).

Poor parental mental health is one of the main risk factors for disrupted parent-infant interactions and may negatively affect bonding and attachment (24). Postpartum depression symptoms diminish the quality of bonding (25–28). However, there are inconsistencies in respect to anxiety and bonding. Namely, some studies showed that anxiety is associated with poor bonding (29), while other studies showed that this relationship is fully mediated by depression. Moreover, once depression was controlled for, anxiety was not associated with poor bonding anymore (28, 30). Nevertheless, one study showed that anxiety was associated with improved mother-infant bonding (31), which the authors attributed to the increased maternal sensitivity. Although, the other study with mothers with social phobia did not show the difference in sensitivity compared to healthy controls (32). A meta-analysis showed that maternal depression symptoms were associated with diminished sensitivity during the first postpartum year (33). However, inconsistencies concerning postpartum depression and responsiveness are evident, as well. It was shown that postpartum depressive symptoms were associated with lower levels of maternal responsiveness (34, 35), or no association was found (36). However, the latter was established in a small sample of mothers with preterm infants.

There is a lack of studies that examines the complex relationship between maternal mental health, maternal sensitivity and responsiveness, and mother-infant bonding. Furthermore, in previous studies, self-report measures of sensitivity and responsiveness or observation during mother-infant interaction have been applied. Although observation is preferred over the self-report measured, the former can also be jeopardized due to personal bias of observer, the difficulty of coding, and change of behavior in the presence of the observer. On the other hand, objective measures of sensitivity, such as facial expression recognition, has been rarely applied. Nevertheless, several new databases of infant facial expression photographs have been developed (37, 38), which could be used as an objective measure of maternal sensitivity. This kind of measures was proven to be sensitive for maternal mental health, as it was shown that, e.g., mothers with postpartum depression tended to rate negative infant faces more negatively (39). Also, previous studies have mainly addressed the quality of bonding as unidimensional, although measured with the Postpartum Bonding Questionnaire (PBQ) (40), which measures different aspects of bonding difficulties. Thus, the role of parental mental health and sensitivity should be examined concerning varying dimensions of bonding.

Finally, previous research on perinatal mental health problems or parental sensitivity has mainly focused on women, thus unjustifiably neglecting fathers (41, 42). Paternal role in the family functioning has substantially changed over the last several decades, with fathers becoming more involved and engaged nowadays (43). Although mothers are rated as more responsive to their preschool children needs than fathers (44), maternal and paternal sensitive parenting have comparable effects on children's cognitive ability (45). Furthermore, it was found that parental sensitivity was a full mediator between parenting stress and child cognitive abilities and prosocial behavior in both mothers and fathers (46). Also, for parental sensitivity, the vital is the parental ability to mentalize a child's thoughts, emotions, and needs that drive their behavior, the so-called reflective functioning, is essential (47). It was shown that the paternal reflective functioning was associated with their toddler's distress, even after accounting for maternal reflective functioning, and it also buffered the adverse effects of low income (41), thus implying the importance of the father's sensitivity for child development. Furthermore, there is a growing body of literature on paternal mental health in recent years, with a meta-analysis showing that around 8% of fathers have postpartum depression (48). In a recent large study of fathers, several depression profiles emerged with substantial stability from pregnancy to 2 months postpartum, although the depression levels decreased in the perinatal period (49). Another meta-analysis showed similar maternal and paternal depression effects on parenting behavior where depressed parents express less positive and more negative behaviors toward their children (50). Also, postpartum mental health difficulties in fathers extend to anxiety and stress (51), with paternal stress mediating the effect of anxiety on father-infant bonding (51). Parent-infant bonding is similar in mothers and fathers, although fathers report less fear and higher emotional involvement with the newborn in the first days after delivery (52). However, studies that would encompass mental health, sensitivity and responsiveness, and parent-infant bonding in fathers, are still scarce, as well as the studies in mother.

Therefore, this study aimed to examine the mediational role of parental sensitivity and responsiveness in a relationship between parental mental health and parent-infant bonding in both mothers and fathers. Also, we wanted to investigate different aspects of mental health, including depression, anxiety, and stress, as the conceptual analysis (5) pointed out as the affecting factors on maternal sensitivity. Furthermore, we wanted to provide different measures of parental sensitivity, including both objective measures of infant facial expression recognition and a self-report measure of responsiveness concerning various aspects of parent-infant bonding. The hypothesis was that parental sensitivity and responsiveness would mediate the relationship between mental health and parent-infant bonding in mothers and fathers.

## Materials and Methods

### Sample

A sample of mothers (*n* = 427) and fathers (*n* = 170) participated in the study. The inclusion criterium was having an infant of 1–12 months. The sample was predominantly married or cohabiting, highly educated, average to above-average self-reported socioeconomic status and lived in a city (Table 1). Approximately 60% of the sample had the first child.

**Table 1:** Sociodemographic data for the sample of mothers (*n* = 427) and fathers (*n* = 170).

	**Mothers (*n* = 427)**	**Fathers *(n* = 170)**	**Comparison**
	***M (SD)***	***M (SD)***	
**Parental age** (age)	30.80 (4.56)	33.19 (5.63)	***t***_**(595)**_ **=** **4.94, ** ***p*** **=** **0.000**
**Infant age** (months)	6.55 (3.23)	6.15 (3.32)	*t*_(595)_ = −1.34, *p* = 0.172
	***n*** **(%)**	***n*** **(%)**	
**Marital status**			
Married or cohabiting	422 (98.8)	170 (100.0)	$\chi_{\left(1\right)}^{2}$ = 0.85,
Separated/divorced/single	5 (1.2)	0 (0)	*p* = 0.3579
**Education**			
Secondary school	107 (25.1)	54 (31.8)	$\chi_{\left(2\right)}^{2}$ = 3.76, *p* = 0.1529
College	62 (14.5)	27 (15.9)	
University or higher	258 (60.4)	89 (52.3)	
**Socioeconomic status**			
Below average	54 (12.7)	21 (12.4)	$\chi_{\left(2\right)}^{2}$ = 0.60,
Average	214 (50.1)	80 (47.0)	*p* = 0.7420
Above average	159 (37.2)	69 (40.6)	
**Place of living**			
Village	74 (17.3)	29 (17.1)	$\chi_{\left(2\right)}^{2}$**=7.57**,
City (<100,000 citizens)	155 (36.3)	43 (25.3)	*p* = **0.0228**
City (more than 100,000 citizens)	198 (46.4)	98 (57.6)	
**Number of children**			
One	252 (59.0)	105 (61.8)	$\chi_{\left(2\right)}^{2}$ = 1.69,
Two	119 (27.9)	39 (22.9)	*p* = 0.4296
Three or more	56 (13.1)	26 (15.3)	
**Psychiatric heredity** ^a^	55 (12.9)	13 (7.7)	$\chi_{\left(1\right)}^{2}$ = 2.80, *p* = 0.0942
**Psychiatric treatment** ^a^	26 (6.1)	3 (1.8)	$\chi_{\left(2\right)}^{1}$ **=** **4.03, ** ***p*** **=** **0.0447**

a *Answer “yes”. Bold font indicates statistical significance*.

The sample of mothers and fathers did not differ in marital status, education level, socioeconomic status, number of children, and psychiatric heredity (Table 1). However, mothers were on average 2.5 years younger than fathers [*M*_mothers_ = 30.80, *M*_fathers_ = 33.19, *t*_(595)_ = 4.94, *p* = 0.000] and less mothers than fathers were from the city larger than 100,000 citizens [mothers: 46.4%, fathers 57.6%, χ${}_{\left(2\right)}^{2}$ = 7.57, *p* = 0.0228]. Also, more mothers reported psychiatric treatment during lifetime [mothers: 6.1%, fathers 1.8%, χ${}_{\left(1\right)}^{2}$ = 4.03, *p* = 0.0447].

### Instruments

*Edinburgh Postnatal Depression Scale* [EPDS; (53)] is a self-report measure of depression symptoms after childbirth. It consists of 10 items with four options different for each item, rated from 0 to 3, out of which seven items are reversely scored. The total possible score ranges from 0 to 30. A higher score indicates a higher level of depression symptoms, and the recent individual patient data meta-analysis established 11 as a cut-off score (54). The EPDS was previously translated and validated in the Croatian perinatal population with a one-factor structure and Cronbach α = 0.86 (55). In the current study, McDonald's ω coefficient was 0.86, respectively.

*Depression, Anxiety, and Stress Scale* [DASS-21; (56)] is a self-report measure with three subscales for depression, anxiety and stress symptoms presented during the previous week. Each item was rated on a four-point scale (0—*Did not apply to me at all* to 3—*Applied to me very much or most of the time*). The scale was translated to Croatian (57). In the current study, a short version with 21 items (7 per subscale) was used, where the final score for each subscale is multiplied by 2 to be comparable to the full scale with a possible range from 0 to 42 where a higher score indicates a higher level of symptoms (56). The anxiety and stress subscales were used in the current study, with McDonald's ω coefficient of 0.84 and 0.88, respectively.

*Maternal Infant Responsiveness Instrument* [MIRI; (36, 58)] is a self-report measure of maternal responsiveness to the infant cues and perception of infant's response. The MIRI consists of 22 items rated on a 5-point scale (1—*strongly disagree* to 5—*strongly agree*), where a higher score indicates higher responsiveness. Six items are reversely scored. In the original study, a total score was calculated as a unidimensional construct, and the Cronbach's was α = 0.87–0.89 (36, 58). The MIRI was previously also administered in fathers, with = 0.88 (59). In the current study, to be comparable to both mothers and fathers, we excluded three items referring to the feeding items (e.g., *I believe I know when my baby wants me to feed him/her*). The CFA showed poor fit with the one-factor model [χ${}_{\left(152\right)}^{2}$ = 1624.12, *p* < 0.001; χ^2^/*df* = 10.69, RMSEA = 0.127, SRMR = 0.100, CFI = 0.803]. Therefore, exploratory factor analysis was performed where the scree plot indicated two factors: positively framed items loaded onto one factor (Responsiveness) and reversely coded items loaded on the second (Non-responsiveness). The CFA was re-run testing the two model with better fit [χ${}_{\left(151\right)}^{2}$ = 857.05, *p* < 0.001; χ^2^/*df* = 5.68, RMSEA = 0.088, SRMR = 0.064, CFI = 0.905] and showed non-significant correlation between the subscales (*r* = −0.03, *p* = 0.4850). The score on the Responsiveness (13 items) and Non-responsiveness subscale (6 items) could range from 13 to 65 and 6 to 30, respectively. Items on the Non-responsiveness scale remained reversely coded. Hence, a higher score on this subscale indicates a higher Non-Responsiveness (exemplary item: *I believe my baby wants me to touch her/him too often*). The McDonald's ω coefficient of internal consistency + was 0.96 for the Responsiveness and 0.77 for the Non-responsiveness.

*City Infant Face Database* [CIFD; (38)] is a set of 154 black-and-white photographs of infant emotional expressions. Photos were collected from infants varied in sex, age (1–12 months), and cultural background where infants express different emotional states, from positive (smiling and laughing), neutral to negative (sad, angry, scared etc.). In this study, we used a previous selection of 139 photographs validated in the sample of Croatian mothers, fathers, and students (60). In the current study, each participant rated the infant expression on 20 randomly chosen photographs (*negative, neutral*, or *positive*). The correct answer was scored with 1 point, so the total possible score ranged from 0 to 20.

*Postpartum Bonding Questionnaire* [PBQ; (40)] is a self-report measure of difficulties in the maternal-infant relationship and has been validated in the sample of mothers with different forms of maternal-infant disorders. The PBQ has 25 items rated on a 6-point scale (0—*never* to 5—*always*), with several reversely scored items, where a higher score indicates more disturbed bonding. Four subscales measure General Factor (12 items), Rejection and pathological anger (7 items), Anxiety about infant (4 items), and Incipient abuse (2 items) (40, 61). The Cronbach's α of the four factors ranged from 0.35 to 0.75 and was 0.78 for the total scale (40). The PBQ was validated in a large sample of Croatian mothers and fathers, where modified 20-item scale showed the excellent fit of both three-factor and one-factor structure: Impaired bonding (10 items, α = 0.94), Anxiety about care and maternal distress (6 items, α = 0.81), Lack of enjoyment and affection with infant (4 items, α = 0.77) (62). In the current study, the same three-factor structure was followed. McDonald's ω coefficient of internal consistency was 0.93, 0.94, 0.81, and 0.77 for the total scale, Impaired bonding, Anxiety about care, and Lack of enjoyment, respectively.

*The sociodemographic questionnaire* comprised question on age, marital status, level of education, employment status (before maternity leave for mothers), perceived socioeconomic level, and place of living. Furthermore, psychiatric history was examined. Participants could report a previous episode of depression or changed mood (*no; yes, shorter than 2 weeks; yes, longer than 2 weeks*), receiving psychiatric treatment (*yes, no*), and psychiatric heredity in the family (*yes, no*). A final set of questions referred to the pregnancy and the infant regarding the number of children, having twins from the last pregnancy, the infant age, and sex.

### Procedures

The study was conducted following Helsinki 1964 Declaration. The Ethical Committee of the Catholic University of Croatia granted the ethical approval for the research. This cross-sectional study was conducted online via Google Forms with separate links for mothers and fathers. It was advertised on social networks (Facebook groups for parents) and shared through personal communication. The data were collected from May 2018 to May 2019. Each participant read the informed consent and by clicking the “Next” button gave their consent to participate in the study. It took ~20 min to fill in all the questionnaires.

### Statistical Analysis

Samples of mothers and fathers were compared in sociodemographic and psychological variables using the t-test and χ^2^-test (with Yates' correction when necessary) with SPSS Statistics 21.0 for Windows and GraphPad Prism version 9.0 for χ^2^-test. Correlations between the studied variables were examined using the Pearson r correlation coefficient. The factor structure of the examined constructs was examined by confirmatory factor analysis (CFA) by MPlus 8.2 software or exploratory factor analysis (Principal Axis Factoring) when necessary.

All variables were normally distributed with skewness and kurtosis index (Table 2) within the suggested 3 and 10, respectively (63), except for the parent-infant bonding. Data on Impaired bonding (PBQ1) and Lack of enjoyment and affection with infant (PBQ3) exceeded both skewness index above 3 and kurtosis index above 20, which indicate serious non-normality (63).

**Table 2:** Descriptive data for psychological variables with comparison between mothers (*n* = 427) and fathers (*n* = 170).

	**Possible range**	**Sample**	**Observed range**	***M***	***SD***	**Skewness**	**Kurtosis**	**Comparison**	**Effect size**
1. Depression symptoms	0–30	Mothers	0–24	7.04	4.72	0.88	0.87	***t***_**(595)**_** = −2.99**,	*r =* 0.12
		Fathers	0–20	5.78	4.52	0.93	0.40	*p* =**0.003**	
2. Anxiety	0–42	Mothers	0–34	3.47	5.51	2.37	6.49	*t*_(595)_ = 0.69,	*r =* 0.03
		Fathers	0–32	3.51	5.95	2.47	6.92	*p* =0.945	
3. Stress	0–42	Mothers	0–38	9.18	7.96	0.97	0.79	***t***_**(595)**_** = −2.12**,	*r =* 0.09
		Fathers	0–36	7.65	7.83	1.18	1.12	*p* =**0.035**	
4. Responsiveness	13–65	Mothers	27–65	59.83	8.09	−2.06	3.20	***t***_**(595)**_** = −5.55**,	*r =* 0.22
		Fathers	25–65	55.69	8.53	−1.36	1.46	*p* =**0.000**	
5. Non-responsiveness	6–30	Mothers	6–26	11.04	4.37	0.86	0.07	***t***_**(595)**_**=2.91**,	*r =* 0.12
		Fathers	6–26	12.18	4.14	0.71	0.28	*p* = **0.004**	
6. Facial expression recognition	0–20	Mothers	5–20	16.52	2.52	−1.59	3.42	*t*_(595)_ = 0.45,	*r =* 0.02
		Fathers	6–20	16.62	2.45	−1.72	3.99	*p* = 0.655	
7. PBQ 1	0–50	Mothers	0–50	2.78	6.12	5.25	32.15	*t*_(595)_ = −0.63,	*r =* 0.03
		Fathers	0–50	2.41	6.98	5.46	31.54	*p* = 0.527	
8. PBQ 2	0–30	Mothers	0–28	4.81	4.14	1.96	6.55	*t*_(595)_ = 0.34,	*r =* 0.01
		Fathers	0–30	4.94	4.69	2.56	9.91	*p* = 0.737	
9. PBQ 3	0–20	Mothers	0–20	0.77	1.85	5.35	40.59	***t***_**(595)**_**=2.91**,	*r =* 0.12
		Fathers	0–16	1.35	2.32	3.31	15.14	*p* = **0.004**	
10. PBQ total scale	0–100	Mothers	0–95	8.35	10.58	4.19	24.05	*t*_(595)_ = 0.35,	*r =* 0.01
		Fathers	0–88	8.70	12.20	4.33	22.81	*p* = 0.730	

*PBQ—Postpartum Bonding Questionnaire: PBQ1—Impaired bonding; PBQ2—Anxiety about care and parental distress; PBQ3—Lack of enjoyment and affection with infant. Bold font indicates statistical significance*.

Path analysis of the associations between parental mental health (depression and anxiety), parental sensitivity, and bonding (three aspects) was performed in MPlus 8.2. The maximum likelihood estimation with robust standard errors—the MLR estimator—was used as this procedure takes into account non-normality induced bias in the standard errors (64, 65). The goodness of fit was evaluated by several indices χ^2^-test, Root Mean Square Error of Approximation (RMSEA), Standardized Root Mean Square Residual (SRMR), and Comparative Fit Index (CFI). Acceptable model fit is indicated when RMSEA and SRMR values are below 0.08, and CFI values are above 0.90 (66), while a very good fit is displayed when the RMSEA is below 0.06, SRMR is below 0.08, and CFI values are above 0.95 (67). Reliability of measures was calculated as the internal consistency via McDonald ω coefficient, as a better alternative to Cronbach α (68) using the OMEGA macro for SPSS (69). Sample size calculation was performed as per the general rule of thumb to have at least 50 participants per variable in the path analysis and to have a medium sample size of 100–200 per group (70). Given that nine variables were examined, at least 450 participants were necessary, which was exceeded with 597 participants, out of which 170 were fathers.

## Results

### Descriptive Data

Descriptive data for all psychological variables is presented in Table 2. A somewhat reduced range was obtained for depression and anxiety in both mothers and fathers. However, 20.8% of mothers and 14.7% of fathers reported depression symptoms above the proposed cut-off of 11 on the EPDS (54). A full range of observed data was obtained for bonding scores, and the almost whole possible range was obtained for responsiveness and facial expression recognition. The scores were compared between mothers and fathers, showing that mothers reported higher depression symptoms, stress, responsiveness, and a lower level of non-responsiveness. On the other hand, fathers expressed more inferior bonding in the Lack of enjoyment and affection with the infant. However, all effects were small (Table 2).

### Associations Between Examined Variable

Very similar patterns of associations were established for mothers and fathers (Table 3). Higher levels of depression symptoms were associated with higher anxiety and stress levels in both samples with modest correlations. Also, in both mothers and fathers, higher parental mental health difficulties (depression, anxiety, and stress) were related to poor bonding but with small correlations. Further, higher levels of mental health difficulties were associated with lower responsiveness and higher non-responsiveness. Facial expression recognition was not related to parental mental health or responsiveness. However, it had a slight negative correlation with non-responsiveness, indicating that poor facial expression recognition was associated with higher non-responsiveness. Also, poor facial expression recognition was related to a Lack of enjoyment and affection with the infant, in mothers only, but with a small correlation.

**Table 3:** Pearson's correlation coefficients between psychological variables in mothers (n = 427, above diagonal) and fathers (n = 170, below diagonal).

	**1**.	**2**.	**3**.	**4**.	**5**.	**6**.	**7**.	**8**.	**9**.
1. Depression symptoms	–	0.57**	0.67**	−0.17**	0.30**	−0.03	0.21**	0.40**	0.14**
2. Anxiety	0.60**	–	0.66**	−0.13**	0.17**	−0.07	0.16**	0.27**	0.09
3. Stress	0.69**	0.70**	–	−0.10	0.21**	0.03	0.21**	0.43**	0.15**
4. Responsiveness	−0.16*	−0.16*	−0.17*	–	0.02	0.07	−0.30**	−0.33**	−0.30**
5. Non-responsiveness	0.37**	0.44**	0.37**	0.00	–	−0.11*	0.09	0.27**	0.02
6. Facial expression recognition	0.08	−0.03	0.11	0.10	0.09	–	0.11*	−0.06	−0.17**
7. PBQ 1	0.30**	0.29**	0.20*	−0.39**	0.18*	−0.08	–	0.70**	0.52**
8. PBQ 2	0.40**	0.31**	0.35**	−0.42**	0.26**	0.05	0.83**	–	0.46**
9. PBQ 3	0.23**	0.29**	0.29**	−0.25**	0.29**	0.06	0.34**	0.35**	–

*p < 0.05 and **p < 0.01. PBQ—Postpartum Bonding Questionnaire: PBQ1—Impaired bonding; PBQ2—Anxiety about care and parental distress; PBQ3—Lack of enjoyment and affection with infant.

### Parental Sensitivity and Responsiveness as Mediators

The model of parental sensitivity as a mediator between parental mental health and bonding was tested. Depression symptoms, anxiety, and stress were entered as predictors; responsiveness, non-responsiveness, and facial expression recognition were entered as mediators; and three aspects of bonding were entered as the outcome. All possible direct and indirect effects were defined in the model. The model was saturated with excellent fit to the data [χ${}_{\left(6\right)}^{2}$ = 14.47, *p* = 0.0248; χ^2^/*df* = 2.41, RMSEA = 0.069, SRMR = 0.021, CFI = 0.989].

**Figure 1:**
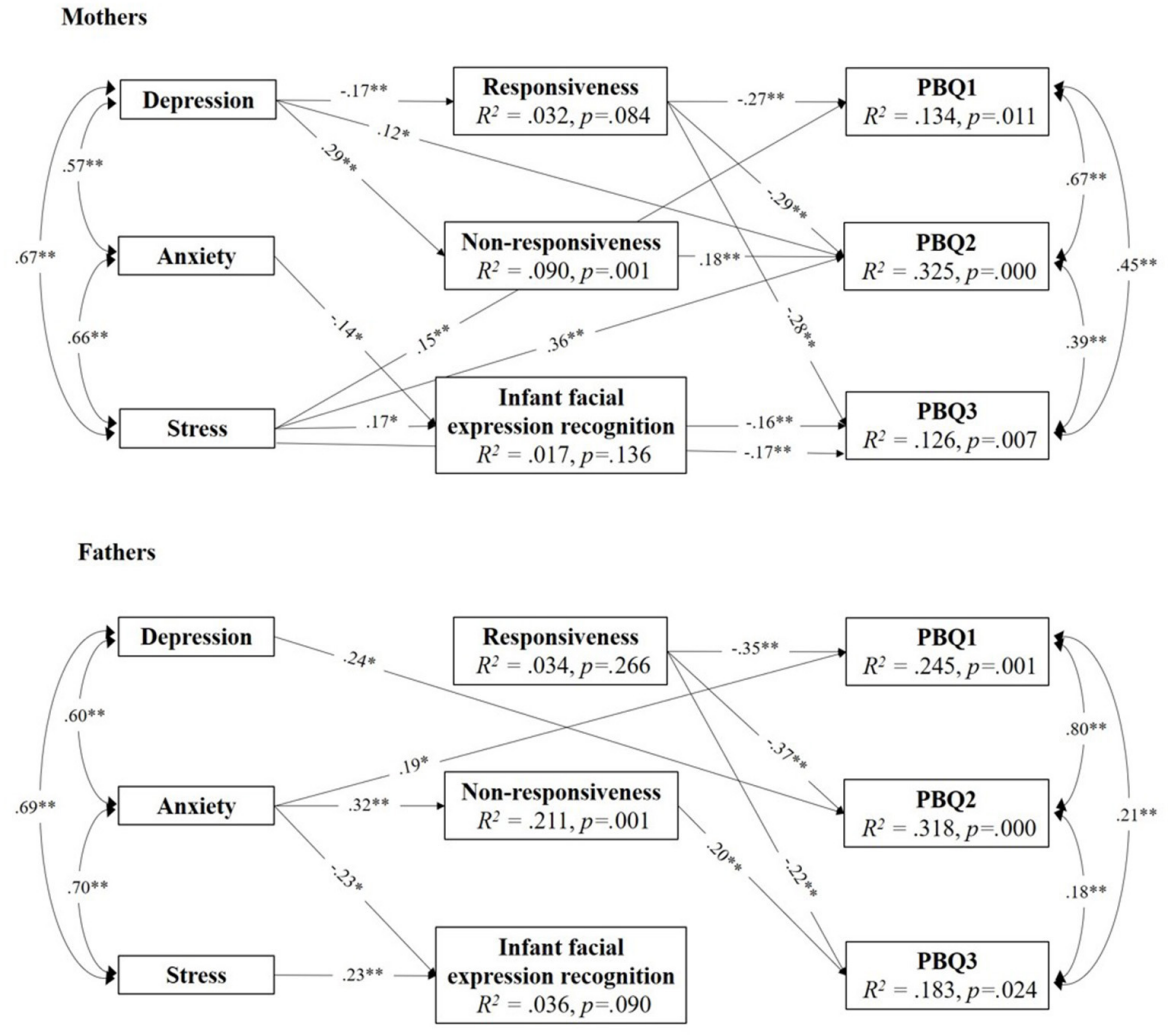
Model paths predicting parent-infant bonding from parental mental health via parental sensitivity and responsiveness in mothers and fathers. Only significant paths are presented. All coefficients are standardized. *p < 0.05 and **p < 0.01.

The parental sex was examined as the moderator in the model. This was tested with the nested model with specified parameters set to be equal between mothers and the fathers [χ${}_{\left(48\right)}^{2}$ = 117.77, *p* < 0.0001; χ^2^/*df* = 2.45, RMSEA = 0.070, SRMR = 0.062, CFI = 0.906]. This model was significantly different from the initial model [Satorra-Bentler Scaled χ^2^ difference was SBS- χ${}_{\left(42\right)}^{2}$ = 103.26, *p* < 0.0001; CD = 1.1766], indicating that the parental sex was a significant moderator. Thus, different paths were established in mothers and fathers (Figure 1).

In mothers, responsiveness was a significant mediator between postpartum depression symptoms and all three bonding dimensions (Table 4). Namely, higher levels of depression symptoms were associated with lower levels of responsiveness, which was, in turn, associated with Impaired bonding, Anxiety about care and maternal distress, and Lack of enjoyment and affection with the infant. Furthermore, non-responsiveness was a significant mediator between maternal depression and one aspect of bonding. More specifically, higher levels of depression were associated with higher levels of non-responsiveness, which was, in turn, related to poor bonding concerning Anxiety about care and maternal distress.

**Table 4:** Model estimates of multigroup path analysis: Depression symptoms and anxiety on bonding via responsiveness and facial expression recognition (*N* = 603).

	**Mothers**			**Fathers**		
	**Path estimates**	**SE**	***p***	**Path estimates**	**SE**	***p***
**Indirect effects via responsiveness**						
Depression → PBQ1	**0.05**	**0.02**	**0.038**	0.02	0.04	0.592
Depression → PBQ2	**0.05**	**0.02**	**0.020**	0.02	0.04	0.592
Depression → PBQ3	**0.05**	**0.02**	**0.043**	0.01	0.03	0.603
Anxiety → PBQ1	0.02	0.02	0.340	0.02	0.04	0.527
Anxiety → PBQ2	0.02	0.02	0.329	0.03	0.04	0.529
Anxiety → PBQ3	0.02	0.02	0.339	0.02	0.03	0.532
Stress → PBQ1	−0.02	0.02	0.377	0.03	0.04	0.528
Stress → PBQ2	−0.02	0.02	0.365	0.03	0.05	0.523
Stress → PBQ3	−0.02	0.02	0.372	0.02	0.03	0.526
**Indirect effects via non-responsiveness**						
Depression → PBQ1	0.01	0.02	0.386	0.01	0.02	0.541
Depression → PBQ2	**0.05**	**0.02**	**0.004**	0.02	0.02	0.322
Depression → PBQ3	−0.01	0.01	0.664	0.03	0.03	0.211
Anxiety → PBQ1	0.00	0.00	0.908	0.02	0.04	0.489
Anxiety → PBQ2	−0.00	0.01	0.908	0.04	0.03	0.186
Anxiety → PBQ3	0.00	0.00	0.911	**0.07**	**0.03**	**0.034**
Stress → PBQ1	0.00	0.00	0.788	0.00	0.01	0.678
Stress → PBQ2	0.00	0.02	0.775	0.01	0.01	0.666
Stress → PBQ3	0.00	0.00	0.811	0.01	0.02	0.669
**Indirect effects via facial expression recognition (CIFD)**						
Depression → PBQ1	0.01	0.01	0.468	−0.00	0.01	0.652
Depression → PBQ2	0.00	0.00	0.615	0.00	0.01	0.639
Depression → PBQ3	0.01	0.01	0.387	0.00	0.01	0.666
Anxiety → PBQ1	0.01	0.01	0.313	0.01	0.01	0.494
Anxiety → PBQ2	0.01	0.01	0.564	−0.01	0.02	0.474
Anxiety → PBQ3	0.02	0.01	0.074	−0.01	0.02	0.473
Stress → PBQ1	−0.02	0.01	0.286	−0.01	0.02	0.526
Stress → PBQ2	−0.01	0.01	0.552	0.01	0.02	0.450
Stress → PBQ3	−0.03	0.02	0.077	0.01	0.02	0.448

*Standardized coefficients are presented. SE, standard error; PBQ—Postpartum Bonding Questionnaire: PBQ1—Impaired bonding; PBQ2—Anxiety about care and parental distress; PBQ3—Lack of enjoyment and affection with infant. Bold font indicates statistical significance*.

In fathers, the only significant indirect path was for non-responsiveness. Higher levels of anxiety were associated with higher levels of non-responsiveness, which was, in turn, related to poor father-infant bonding concerning Lack of enjoyment.

Finally, facial expression recognition did not mediate mental health and bonding in mothers or fathers. Nevertheless, it did directly affect bonding in mothers so that mothers who were less accurate at recognition reported higher levels of Lack of enjoyment and affection with baby. Also, even though anxiety and stress did not correlate with infant facial expression recognition in mothers or fathers, in the model, these direct effects were significant, indicating possible suppressor effect (71, 72). Direct effects from parental mental health on all three dimensions of bonding were established (Figure 1). However, it is interesting to note that anxiety did not directly affect bonding concerning the Anxiety about care. Also, responsiveness had a direct effect on all dimensions of bonding, both in mothers and fathers.

## Discussion

There was a lack of studies looking into the role of maternal sensitivity for mother-to-infant bonding in the literature, and even more, there was a neglect of fathers. Therefore, this study aimed to examine parental sensitivity as a mediator in the relationship between parental mental health and parent-infant bonding in both mothers and fathers. The model had a good fit to the data, and parental responsiveness was a significant mediator between postpartum mental health and bonding quality. However, different paths were established for mothers and fathers, which will be discussed further.

First, parental sensitivity in the current study was measured by a self-report measure of responsiveness as one aspect of sensitivity (5) and an objective measure of infant facial expression recognition. Also, before going further, it should be noted that the Maternal Infant Responsiveness Instrument was previously used as a unidimensional measure (34–36, 58, 59) without questioning its factor structure. However, the initial psychometric evaluation in the current study showed a poor fit of the unidimensional model to the data. This secondary finding highlights the need for psychometric testing of instruments at each administration. Namely, psychometric properties are not fixed characteristics of the instrument, as they also reflect the sample characteristics and administration circumstances (73). The two-factor structure of the MIRI had a better fit and resulted in subscales of responsiveness and non-responsiveness. These two were mutually uncorrelated, indicating that the non-responsiveness subscale is not a mere negative pole of responsiveness. Moreover, non-responsiveness taps different responsiveness aspects, reflecting fear of taking care of the infant and appraisals of the infant as being too demanding. Furthermore, it was interesting that these two subscales had a unique role in the relationship between mental health and bonding in mothers and fathers.

In mothers, responsiveness was a significant mediator between depression symptoms and bonding. Higher levels of depression symptoms were associated with lower levels of responsiveness, which was, in turn, related to poor bonding on all three dimensions, i.e., Impaired bonding, Anxiety about the care, and Lack of enjoyment with the infant. In fathers, responsiveness was not a significant mediator between mental health and bonding. However, non-responsiveness was a significant mediator both for mothers and fathers. Despite specific differences in the patterns of mediational pathways, we can summarize that both for mothers and fathers, (non)responsiveness has an important role in the shape of parent-infant bonding.

These findings are somewhat difficult to relate to previous research on bonding, as these constructs have not been examined all together in a mediational model, especially not in fathers. However, previous studies demonstrated an adverse effect of maternal depression symptoms on maternal sensitivity (33) and maternal responsiveness (34). On the other hand, it is not easy to compare findings on parental sensitivity and parent-infant bonding, as previous studies have mainly investigated maternal sensitivity observationally with infant-mother attachment (1, 6, 7). The same goes for examining the relationship between responsiveness and mother-infant bonding. However, Tester-Jones et al. (35) did investigate depression symptoms, maternal responsiveness, and bonding, but they did not relate these constructs in the same model but on the bivariate level. They did show that maternal depression was associated with lower levels of responsiveness and bonding, and these relationships were mediated by infant temperament.

On the other hand, another study did not show an association between maternal depressive symptoms and responsiveness but found a more dominant role of stress for responsiveness (36). However, the latter finding comes from a small sample of mothers with preterm babies who have specific childbirth and postpartum experience. It is known that mothers with preterm delivery are at higher risk of posttraumatic stress disorder following birth (74), which is, in turn, associated with impaired bonding in mothers (75).

The ability to recognize infant facial expressions was previously suggested to reflect maternal sensitivity (38). However, the mediational role of infant facial expression recognition was not established in the current study, either for mothers or fathers. It was expected that depression symptoms would be associated with facial expression recognition, but this was not evident. This finding was unexpected as previous studies showed that depressed mothers were less likely to identify happy infant faces (76) and rated negative infant faces more negatively (39). Different attentional processing of positive and negative infant emotions associated with depression symptoms was evident even during pregnancy (77, 78). A similar effect of depression was demonstrated in fathers, as well. A recent study showed that depressed fathers recognized happy faces with more difficulty but negative faces more easily, which, in turn, affected negatively on the father-infant interaction (79). On the other hand, some studies did not show attentional bias toward negative infant faces in mothers with affective disorders (80). So, the infant facial expressions recognition remains to be demonstrated as a measure of maternal sensitivity to infant's cues and its role in predicting parent-infant bonding.

The findings of this study have several implications for clinical practice. First, the study highlights the need for screening for a wide range of mental health difficulties. In addition to depression symptoms that most screening attempts are focused on (81), anxiety and stress also contributed to parental sensitivity and parent-infant bonding. Also, the screening should be applied to both mothers and fathers (82, 83). Because of the contributing effect of the partner depression (84), both parents can get into a vicious circle of depression, where a parent has a higher probability of developing depression symptoms if their partner also shows depression symptoms. Also, fathers should be provided with the same opportunities in the (prenatal) classes as mothers have to learn about newborn care, parenting sensitivity, and parent-infant bonding. Bonding between fathers and infants is a process that develops over the first year of the infant's life, as shown in the meta-synthesis of paternal experiences (43). The process progresses by getting to know the infant and having physical contact and interaction with the infant, which is especially rewarding for fathers. Therefore, courses for paternal engagement and enhancement in bonding should encourage fathers to take care of infants, play with them, or simply hold them. As they may feel the lack of knowledge and skills in infant care, they should be taught about this in (prenatal) classes and supported by their spouses, as fathers found their partners' support very encouraging (43). Particular focus should be on fathers whose infants are breastfed, as they may feel excluded and may need some additional time to catch up with their infant. Also, concerning the parental role in fathers, future studies should shift more from mere involvement, i.e., quantity, to the father-child relationship quality (42). Furthermore, sensitive parenting should be promoted to ensure a safe environment that is supportive and stimulating for the child development. Parents should comfort the child and provide a secure base for their exploration and autonomy (41, 42).

Several limitations of the study should be discussed. First, this sample of mothers and fathers was a non-clinical sample. Therefore, other possible conclusions could be withdrawn if the sample included parents with clinical depression, anxiety, or a bonding disorder. Nevertheless, at least one part of the parents from the sample struggled with depression symptoms, as one in five mothers and one in six fathers reported clinically significant depression symptoms. Furthermore, the sample was recruited online via social network groups for parents, so one can argue that this sample is self-restricted. Indeed, the sample was urban, highly educated; almost all parents were married or cohabiting, with the majority reporting average to above-average socioeconomic status. As they have decided to participate in this study, they were probably interested in content about parenting and more engaged in their parental role. The sample of fathers was smaller than the sample of mothers; therefore, future studies would benefit from including the larger samples of fathers in order to replicate these findings. Also, the cross-sectional design was applied so one can speculate that different directions of associations could work as well. For instance, Brockington et al. (40) highlighted that depression in mothers could be caused or exaggerated by bonding problems. Although the model has a solid theoretical background, it was not previously tested for bonding, and future studies should confirm the model in longitudinal studies. Maternal interpersonal sensitivity measured during pregnancy was a stronger predictor of the mother-infant interaction quality than perinatal depressiveness (85), so it would be beneficial to measure maternal sensitivity even during pregnancy.

In this study, the role of anxiety for responsiveness and bonding was found only in fathers. However, it should be noted that a general measure of anxiety (DASS-21) was used in this study, which mainly covers somatic symptoms. Recent research has shown that anxiety specific for the postpartum period has a predictive value for bonding over general measures (86). Future studies could benefit from applying specific measures of anxiety that grasp the parental perinatal experience with more focus. Also, it should be noted that bonding was measured up to 12 months of the infant's age (with a mean at 6 months). However, the PBQ was designed for use in the early postpartum period (40, 61), and it has been mostly used and validated within the first 3 months after childbirth (87–90). Nevertheless, some other studies applied the PBQ within the first postpartum year [e.g., (91, 92)]. Still, the factor structure and reliability across the first year postpartum should be examined in future studies. Furthermore, the infant facial recognition task included the recognition of unknown infant faces. As postpartum mothers have specific dopaminergic reward-related brain network activation when viewing their infants compared to unknown infant's faces (93), future studies should preferably include expressions of their infant.

Finally, it should be noted that the examined set of variables explained up to 32% of the parent-infant bonding variance. It means that two-thirds of the variance remains unexplained, and future studies should include other variables into the model. A recent cross-sectional study showed the interrelation of maternal mental health and bonding with perceived infant temperament (94). Infant temperament has been shown to affect the parent-infant bonding in a prospective study in mothers and father (95, 96). It also mediates the relationship between maternal depression and responsiveness (35) and might have a more substantial effect on infant-mother attachment than maternal sensitivity (97).

Also, previous studies have established the association between breastfeeding and maternal sensitivity. Longer breastfeeding was associated with higher maternal sensitive responsiveness levels during infancy (98) and even increased maternal sensitivity in middle childhood (99). Nevertheless, in the current study, different infant feeding methods were not considered as we wanted to test the same model in both mothers and fathers. The study's strength is including both parents, and further studies should focus on fathers in more depth. Also, future studies would benefit from pairing mothers and fathers so the dyadic relationships within the couple can be examined. A dyadic analysis on first-time parents revealed that postpartum depression levels are affected by own anxiety and parenting stress and partners' depression in both mothers and fathers (84). A recent study showed that mother-infant bonding contributes to father-infant bonding (51), and dyadic relationships of parental mental health and bonding should be further examined. Finally, some more stable characteristics, such as life satisfaction and self-esteem, seem to be more important predictors of maternal responsiveness (58), so the range of examined variables could be expanded.

To conclude, the current study showed that responsiveness has an important mediational role in the relationship between parental mental health and parent-infant bonding, both for mothers and fathers. This finding fits into Shin et al.'s (5) conceptual analysis of maternal sensitivity affected by maternal mental health. The model can be extended to apply not only for attachment as an infant-to-mother relationship but also to bonding as a mother-to-infant relationship and for fathers. However, theoretical and empirical work is needed to provide a solid theoretical basis for future studies on parent-infant bonding. It could have a crucial impact on developing interventions for parents and infants to alleviate mental health problems and their reflection on the bonding issues. A promising early intervention for reinforcing maternal sensitivity, especially in women with psychosocial vulnerability, has been tested recently (100). Future studies should continue developing such programs to help parents enjoy this transition to parenthood and provide safe and warm family relations for the growth of the child.

## Data Availability Statement

The raw data supporting the conclusions of this article will be made available by the authors, without undue reservation.

## Ethics Statement

The studies involving human participants were reviewed and approved by Ethical Committee of the Catholic University of Croatia. The patients/participants provided their written informed consent to participate in this study.

## Author Contributions

SNR devised the main conceptual idea, supervised the project, performed the analysis, and wrote the manuscript.

## Conflict of Interest

The author declares that the research was conducted in the absence of any commercial or financial relationships that could be construed as a potential conflict of interest.

## Publisher's Note

All claims expressed in this article are solely those of the authors and do not necessarily represent those of their affiliated organizations, or those of the publisher, the editors and the reviewers. Any product that may be evaluated in this article, or claim that may be made by its manufacturer, is not guaranteed or endorsed by the publisher.
